# Factors associated with postnatal care utilisation in Rwanda: A secondary analysis of 2010 Demographic and Health Survey data

**DOI:** 10.1186/s12884-016-0913-0

**Published:** 2016-05-31

**Authors:** Bernard N. Rwabufigiri, Judith Mukamurigo, Dana R. Thomson, Bethany L. Hedt-Gautier, Jean Paul S. Semasaka

**Affiliations:** School of Public Health, College of Medicine and Health Sciences, University of Rwanda, Kigali, Rwanda; Department of Global Health and Social Medicine, Harvard Medical School, Boston, MA USA; Partners In Health–Rwanda, Rwinkwavu, Rwanda

**Keywords:** Africa, DHS, PNC

## Abstract

**Background:**

Postnatal care (PNC) in the first seven days is important for preventing morbidity and mortality in mothers and new-borns. Sub-Saharan African countries, which account for 62 % of maternal deaths globally, have made major efforts to increase PNC utilisation, but utilisation rates remains low even in countries like Rwanda where PNC services are universally available for free. This study identifies key socio-economic and demographic factors associated with PNC utilisation in Rwanda to inform improved PNC policies and programs.

**Methods:**

This is a secondary analysis of the 2010 Demographic and Health Survey, a national multi-stage, cross-sectional survey. In bivariate analysis, we used chi-square tests to identify demographic and socio-economic factors associated with PNC utilisation at α = 0.1. Pearson’s R statistic (*r* > 0.5) was used to identify collinear covariates, and to choose which covariate was more strongly associated with PNC utilisation. Manual backward stepwise logistic regression was performed on the remaining covariates to identify key factors associated with PNC utilisation at α = 0.05. All analyses were performed in Stata 13 adjusting for sampling weights, clustering, and stratification.

**Results:**

Of the 2,748 women with a live birth in the last two years who answered question about PNC utilisation, 353 (12.8 %) returned for PNC services within seven days after birth. Three factors were positively associated with PNC use: delivering at a health facility (OR: 2.97; 95 % CI: 2.28–3.87), being married but not involved with one’s own health care decision-making (OR: 1.69; 95 % CI: 1.17, 2.44) compared to being married and involved; and being in the second (OR: 1.46; 95 % CI: 1.01–2.09) or richest wealth quintile (OR: 2.04; 95 % CI: 1.27–3.29) compared to the poorest. Mother’s older age at delivery was negatively associated with PNC use (20–29 – OR: 0.51, 95 % CI: 0.29–0.87; 30–39 – OR: 0.47, 95 % CI: 0.27–0.83; 40–49 – OR: 0.32, 95 % CI: 0.16–0.64).

**Conclusions:**

Low PNC utilisation in Rwanda appears to be a universal problem though older age and poverty are further barriers to PNC utilisation. A recent change in the provision of BCG vaccination to new-borns might promote widespread PNC utilisation. We further recommend targeted campaigns to older mothers and poorest mothers, focusing on perceptions of health system quality, cultural beliefs, and pregnancy risks.

## Background

The postnatal period, defined as the first six weeks after birth, is an important time in the life of the mother and child [1]. Care received during this period is important for preventing morbidity and mortality in mothers and new-borns [2]. In 2013, more than 289,000 maternal deaths occurred globally, with 62 % occurring in sub-Saharan Africa alone [3]. As a result, millennium development goals 4 and 5 focus on reduction of child mortality by two-thirds, and reduction of maternal mortality by three-quarters between 1990 and 2015, respectively [4, 5]. The United Nations has set targets for 2030 to further reduce child and maternal mortality [6].

The first week postpartum is especially critical in the life of the mother and the baby because half of all postnatal maternal deaths and 850,000 neonatal deaths happen during this window [7, 8]. The most common causes of maternal mortality in Africa are postpartum haemorrhage (33.9 %), hypertensive disorders (9.1 %) and postpartum sepsis (9.1 %) [9]. For new-borns, the most common causes of death are infection (26 %), low birth weight (28 %), and asphyxia (23 %) [7]. Almost all causes of maternal and neonatal mortality are preventable with good postnatal care (PNC) within the first week [10].

By emphasizing maternal and child health services, countries in sub-Saharan Africa brought the percent of pregnant women accessing antenatal care (ANC) from 66 % to 79 % between 2000 and 2009 [11]. Despite achievements in linking women to ANC services, PNC utilisation is still very low. For example, 98 % of pregnant women in Tanzania and Rwanda attend at least one ANC visit, while only 31 % and 18 % of women receive PNC within two days of delivery in those countries, respectively [12, 13].

Reported PNC figures in national household surveys assume that women who delivered at a facility were provided a post-natal check-up before discharge; however, anecdotal evidence observed by co-author JPS, a practicing clinician in Rwanda, suggests that full PNC services are not consistently or fully provided at delivery discharge. Furthermore, PNC for new-borns is often lower and not well tracked; for example, Tanzania does not track new-born PNC, and it is only 5 % in Rwanda [12, 13].

This paper focuses on Rwanda where strong governmental support for maternal and child health interventions has recently resulted in massive reductions in child and maternal mortality [14]. The Rwanda Ministry of Health has introduced interventions that could improve PNC utilisation including training of health care providers in emergency obstetric and new-born care, linking women and neonates in need of care to community health workers (CHWs) via SMS, community performance-based financing to motivate CHWs to support mothers and babies, free maternal services (ANC, PNC and family planning) and incentives to women who utilized these services [14]. A key barrier to PNC in Rwanda is the cultural practice of *kwita izina* which discourages both the mother and new-born from leaving the house until the new-born is named at eight days. The Rwandan Ministry of Health recently set a target to have 50 % of women and new-borns seen by a medical professional in the first seven days of life [15].

The goal of this study is to identify factors associated with utilisation of PNC to inform future PNC policies and programs in Rwanda.

## Methods

### Study setting

The Rwandan health system is decentralised with three main levels of care: health centre, district hospital, and referral hospital. Health centres provide preventative and basic curative services including child immunizations and treatment of common child illnesses; monitor child nutrition, family planning, antenatal, delivery and postnatal care; and refer complex cases to district hospitals [15]. In Rwanda, PNC services are primarily offered in health centres and are offered to all post-partum women free of charge. In addition, one dedicated CHW based in each village provides free contraceptives (condoms, oral, injectable, and CycleBeads), identifies and refers women and new-borns with danger signs to health facilities, accompanies women in labour to health facilities, and encourages early postnatal check-ups at health facilities for mothers and new-borns.

### Study design, data collection and analysis

This is a secondary data analysis of the 2010 Rwanda Demographic and Health Survey (RDHS). The 2010 RDHS is a cross-sectional survey that uses multistage cluster sampling of villages and households, with stratification by all 30 districts. Of the 13,671 women age 15 to 49 years who consented to participate in the survey, 2,748 had a live birth in the last two years and responded to questions about that pregnancy and delivery. Births in the last two years were studied to maintain a consistent definition of PNC with the RDHS [12] and to measure recent trends in PNC.

The study identified factors related to use of PNC by using a multivariate logistic regression model. We first tested bivariate associations using chi-square tests between PNC utilisation and demographic and socio-economic factors that were identified by a conceptual framework and available in the 2010 RDHS. We define PNC utilisation as returning to a health facility after delivery for a full PNC check-up. Variables that had a marginally significant relationship with PNC utilisation (*p* < 0.1) were considered for the final model. Next we tested for collinearity among marginally significant covariates using Pearson’s R coefficient. When variables were collinear (*r* > 0.5), we kept the one that was more strongly associated with PNC utilisation. We used manual backward stepwise regression to identify factors that had a relationship with PNC utilisation, keeping in the final model factors that were significant at *p* < 0.05. All models included place of delivery, urban/rural residence, mother's age at delivery, and household wealth index.

All analyses applied sampling weights and adjusted for clustering and stratification of observations. Descriptive results are reported as percentages and frequencies, and bivariate and multivariate results are reported as odds ratios with 95 % confidence intervals. All analyses were performed in Stata/SE version 12 software.

### Ethics statement

This study is a secondary data analysis of the RDHS which is publically available, and permission was received from MEASURE DHS Data Archive at ICF International to conduct this study. A private space was secured and the respondent's informed verbal consent was obtained before the interview. Ethical review for data collection and de-identification protocols were granted by the Rwanda National Ethics Committee and the MEASURE DHS Project. Further, this study was registered and approved by the University of Rwanda-College of Medicine and Health Sciences-School of Public Health Internal Review Board (031/UR/CMHS/SPH/2014).

## Results

Of the 2,748 women with a live birth in the last two years who answered question about PNC utilisation, 353 (12.8 %) returned for PNC services within seven days of birth. In bivariate analysis, PNC utilisation was associated with child’s birth order (*p* < 0.001), but not significantly associated with any of the other child-related factors (Table 1). For the mother-related factors, the following items were significant: education level (*p* < 0.001), insurance status (*p* = 0.002), wanted the last pregnancy (*p* = 0.010), received tetanus injection before birth (*p* < 0.001), number of antenatal visits (*p* < 0.001), assisted delivery (*p* < 0.001), Caesarean section delivery (*p* < 0.001), age at delivery (*p* < 0.001), health facility delivery (*p* < 0.001), if getting money is a barrier to health care (*p* = 0.046), and her involvement in her own health care decisions (*p* = 0.010). Household-related factors significantly associated with receiving PNC included: living in a rural area (*p* = 0.004), living in Northern or Southern province (*p* < 0.001), and household wealth status (*p* < 0.001) (Table 1). We excluded birth order and assisted delivery from the multivariate analysis because they were collinear with place of delivery and mother’s age at delivery, respectively, and less associated with PNC utilisation.

**Table 1:** Demographic and socio-economic characteristics of study population

	No PNC		PNC Use		Chi-Square *p*-value^a^
	Weighted N	%	Weighted N	%	
**Child-related factors**					
Child's sex					0.653
Boy	1223	51.0	185	52.4	
Girl	1173	49.0	168	47.6	
Size at birth^b^					0.435
Small size	353	14.8	58	16.4	
Normal or larger size	2028	85.2	294	83.6	
Birth order number					**<0.001**
One	345	14.4	99	28.0	
Two	414	17.3	80	22.7	
Three	349	14.6	58	16.4	
Four and more	1287	53.8	116	32.9	
**Mother-related factors**					
Mother's age at delivery					**<0.001**
<20 years	97	4.1	31	8.9	
20–29 years	1155	48.2	186	52.8	
30–39 years	853	35.6	112	31.8	
40–49 years	290	12.1	23	6.5	
Marital status, and decision about woman’s health care^b^					**0.010**
Never married, divorced, separated, widowed	434	18.1	57	16.1	
Married or partnered, woman not involved in decisions	529	22.1	103	29.3	
Married or partnered, woman involved in decisions	1427	59.7	192	59.1	
Mother's education					**<0.001**
No education	581	24.2	51	14.5	
Primary	1655	69.1	255	72.3	
Secondary/higher	160	6.7	47	13.3	
Mother's employment^b^					0.200
Not working	208	8.7	38	10.8	
Working	2184	91.3	315	89.2	
Wanted last child*					**0.010**
Wanted child	1283	53.6	209	59.3	
Wanted child but later	664	27.7	100	28.3	
Wanted no more children	447	18.7	44	12.5	
Antenatal visits during pregnancy^b^					**<0.001**
Less than four	1698	71.1	206	58.8	
Four and more	691	28.9	145	41.2	
Received tetanus injections before birth*					**<0.001**
No	712	29.9	64	18.1	
Yes	1668	70.1	288	81.9	
Assisted delivery^b^					**<0.001**
No	1628	68.1	141	40.1	
Yes	763	31.9	210	59.9	
Delivery by caesarean section					**<0.001**
No	2113	88.2	286	80.9	
Yes	282	11.8	67	19.1	
Place of delivery					**<0.001**
Not at health facility	1543	64.4	120	33.9	
At health facility	852	35.6	233	66.1	
Mother insured^b^					**0.002**
No	810	33.8	88	24.8	
Yes	1583	66.2	265	75.2	
Getting medical help for self: distance to health facility^b^					0.640
Big problem	761	31.8	108	30.6	
Not a big problem	1632	68.2	245	69.4	
Getting medical help for self: getting money needed for treatment^b^					**0.046**
Big problem	1459	61.0	196	55.5	
Not a big problem	934	39.0	157	44.5	
**Household-related factors**					
Place of residence					**0.004**
Urban	265	11.1	58	16.5	
Rural	2130	88.9	295	83.5	
Wealth index					**<0.001**
Poorest	650	27.1	72	20.5	
Poorer	563	23.5	82	23.3	
Middle	489	20.4	59	16.7	
Richer	397	16.6	60	17	
Richest	297	12.4	80	22.6	
Region					**<0.001**
Kigali city	203	8.5	50	14.2	
South	616	25.7	129	36.5	
West	568	23.7	39	11.1	
North	397	16.6	83	23.5	
East	611	25.5	52	14.7	

^a^ Statistically significant *p* values are in bold

^b^ Missing responses

In the reduced multivariate model, three factors were positively associated with PNC use: delivering at a health facility (OR: 2.97; 95 % CI: 2.28, 3.87), being married but not involved with one’s own health care decision-making (OR: 1.69; 95 % CI: 1.17, 2.44) compared to being married and involved; and being in the second poorest (OR: 1.46; 95 % CI: 1.01, 2.09) and richest wealth quintile (OR: 2.04; 95 % CI: 1.27, 3.29) compared to poorest wealth quintile (Table 2). Mother’s older age at delivery was negatively associated with PNC use; older women had lower odds of using PNC (20 - 29 years - OR: 0.51; 95 % CI: 0.29, 0.87; 30 - 39 years - OR: 0.47; 95 % CI: 0.27, 0.83; 40 - 49 years - OR: 0.32; 95 % CI: 0.16, 0.64, compared less than 20 years) (Table 2). Also women living in Western province (OR: 0.51; 95 % CI: 0.27, 0.95) had lower odds of PNC utilisation compared to residents of Kigali city (Table 2). Urban/rural residence, antenatal care attendance, education status of the mother, and mother’s perception that distance to health facility was a barrier were not associated with PNC utilisation.

**Table 2:** Multivariate associations between post-natal care within 7 days of delivery, and demographic, socioeconomic, and clinic factors in Rwanda, 2010

	Full Model			Reduced Model		
	(*n* weighted=2599)			(*n* weighted=2741)		
	OR^a^	95 % CI^b^	*p*-value^c^	OR^a^	95 % CI^d^	*p*-value^c^
Mother’s age at delivery						
<20	1.00			1.00		
20–29	0.63	[0.37, 1.08]	0.091	0.51	**[0.29, 0.87]**	**0.015**
30–39	0.60	[0.34, 1.06]	0.079	0.47	**[0.27, 0.83]**	**0.010**
40–49	0.45	[0.22, 0.93]	0.030	0.32	**[0.16, 0.64]**	**0.001**
Marital status/Decision in health care						
Never Married/divorced/separated/widowed	1.00			1.00		
Married/partnered, not involved in decision	1.67	[1.12, 2.50]	0.012	1.69	**[1.17, 2.44]**	**0.005**
Married/partnered, involved in decision	1.07	[0.74, 1.53]	0.722	1.11	[0.79, 1.55]	0.525
Mother's education						
No education	1.00					
Primary	1.17	[0.84, 1.61]	0.353			
Secondary/higher	1.24	[0.75, 2.08]	0.401			
Wanted last child						
Wanted child	1.00					
Wanted child but later	1.03	[0.78, 1.35]	0.839			
Wanted no more child	0.89	[0.62, 1.28]	0.534			
Antenatal visits during pregnancy						
Less than four	1.00					
Four and more	1.18	[0.91, 1.53]	0.217			
Received tetanus injections before birth						
No	1.00					
Yes	1.24	[0.88, 1.74]	0.213			
Delivery by caesarean section						
No	1.00					
Yes	0.78	[0.54, 1.11]	0.164			
Place of delivery						
Not at health facility	1.00			1.00		
At health facility	2.80	[2.07, 3.79]	<0.001	2.97	**[2.28, 3.87]**	**<0.001**
Mother insured						
No	1.00					
Yes	1.17	[0.87, 1.58]	0.284			
Getting medical help for self: getting money needed for treatment						
Big problem	1.00					
Not a big problem	0.91	[0.71, 1.15]	0.424			
Place of residence						
Urban	1.00			1.00		
Rural	1.02	[0.64, 1.64]	0.922	1.04	[0.66, 1.62]	0.857
Wealth index						
Poorest	1.00			1.00		
Poorer	1.39	[0.96, 2.01]	0.084	1.46	**[1.01, 2.09]**	**0.040**
Middle	1.10	[0.75, 1.62]	0.632	1.14	[0.78, 1.66]	0.488
Richer	1.37	[0.92, 2.03]	0.120	1.42	[0.97, 2.08]	0.067
Richest	2.07	[1.23, 3.47]	0.005	2.04	**[1.27, 3.29]**	**0.003**
Region						
Kigali city	1.00					
South	1.60	[0.90, 2.84]	0.108	1.53	[0.89, 2.63]	0.119
West	0.54	[0.28, 1.02]	0.058	0.51	**[0.27, 0.95]**	**0.036**
North	1.69	[0.91, 3.14]	0.093	1.57	[0.88, 2.81]	0.125
East	0.69	[0.37, 1.30]	0.250	0.62	[0.34, 1.14]	0.129

^a^ Adjusting for all other covariates

^b^ 95 % confidence interval

^c^ Based on *t*-test

^d^ Statistically significant *p*-values and its 95 % CI are in bold

## Discussion

PNC utilisation in Rwanda is much lower than desired, though we identified several factors that may be associated with utilisation. Engagement with the health system before delivery was not associated with PNC, but engagement with the health system at delivery was. There are mixed results linking ANC and PNC utilisation, in some studies women’s PNC utilisation was associated with previous ANC [16–18], while in other studies it was not [19, 20]. Numerous studies find that delivery at a health facility is associated with PNC utilisation [17, 18, 21, 22]. This may be because women who deliver at a health facility are encouraged to come back within a few days by their health providers, and those women are already familiar with the health facility. Women who deliver at a facility also demonstrated ability to overcome cultural, geographic, financial and other barriers related to health care access. ANC may not have the same effect because ANC services are received much earlier in time.

Our finding that older age was associated with less PNC utilisation is similar to other studies [2, 22]. One hypothesis is that the health system in Rwanda has experienced large improvements in quality in recent years [23], and therefore older women perceive the health system as low quality based on previous bad experiences. The second hypothesis is that older women have grown up with the mentality that pregnancy is not a disease and therefore does not require women to seek care at a health facility during or after pregnancy; this mind-set, which is common in other settings, suggests that only a “weak woman” would need medical care during pregnancy [24]. Young mothers who have grown up with more messaging around ANC, delivering in a facility, and PNC are therefore more likely to take advantage of community health workers and the medical system [25].

We expected financial and geographic barriers to health care to be associated with low PNC utilisation based on findings in Tanzania and Nigeria [19]; however, our findings suggest that financial and geographic barriers are not main issues in Rwanda. Financial barriers to PNC in Rwanda may be mediated by free universal PNC services offered at all health centres nationwide. Since education level and income are highly correlated, universal access to PNC in Rwanda might also mediate educational barriers to maternal health services that are seen in other similar settings where education is associated with ANC delivery or PNC [17–19]. The lack of geographic barriers to PNC may be explained by good coverage of health centres in all sectors (3rd level administrative unit), which might also explain why rural residence was not a risk factor for low PNC utilisation even though rural women in other African countries are less likely to access PNC [17, 18].

We were surprised to find that women who were not involved in their own health care decision-making were more likely to use PNC services than women involved in their health care decision-making. A pervasive theory in public health is that women who are empowered to make health care decisions will use that power to make healthier choices than their husband [26, 27]. This is reinforced by studies that show health decision-making is associated with higher attendance of health care utilisation for the women and her family [28]. However the pattern in Rwanda appears to be different. Another study of maternal health in Rwanda found that women in male-headed households more likely to attend ANC, deliver at a health facility, and seek PNC than women in female-headed households [29]. They hypothesize that male-headed households have greater ability to overcome social and economic barriers to health care than female-headed households.

Several policies and programs may address the universal problem of low PNC utilisation in Rwanda. Previously BCG vaccination was offered before discharge to women who gave birth at a facility; however, a recent change in protocol means that facilities only offer BCG vaccinations on certain days to prevent wastage in open vials. These vaccination visits which occur in the first week after birth are now being used for PNC; this protocol change was witnessed by authors (JPS and JM) at multiple health facilities in urban and rural Rwanda in early 2014. Although the impact is not yet fully measured, the proportion of babies and mothers receiving PNC might increase if facilities have the capacity to perform mass PNC consultations on these days. Linking older women and poorest women into ANC, delivery, and PNC could further improve health outcomes, and therefore information campaigns should target these populations, specially addressing concerns about health system quality, cultural perceptions, and increased risks associated with pregnancy in older women. These targeted campaigns might occur at hospital maternity homes, which are common in rural Africa for women to stay in their final weeks of pregnancy [30]. One such home near Ruli Hospital in Rwanda offers skills training such as basket weaving that can be used by women to generate income after delivery; we recommend adding PNC information and direct services to these types of programs.

There were several limitations to this study. As a cross-sectional survey, we are unable to draw causal conclusions. Furthermore, as a secondary data analysis, some important variables were not available for analysis such as cultural beliefs about when women are allowed to leave the house in the postpartum period, roles of husbands in maternal health decision-making, and perceptions about whether pregnancy is a medical issue warranting clinical visits.

## Conclusion

Low PNC utilisation in Rwanda appears to be a universal problem; other than older age and poverty, few variables appeared to be limiting factors in our analysis. Uptake of PNC services may be widely improved by linking PNC to first BCG vaccination, and special barriers faced by older women and poorer women may be overcome by targeted programs through hospital maternity homes.

## Abbreviations

ANC, antenatal care; BCG, Bacillus Calmette–Guérin; CI, confidence interval; CHW, community health worker; OR, odds ratio; PNC, postnatal care; RDHS, Rwanda Demographic and Health Survey; SMS, short messaging service
